# The Long Noncoding RNA NEAT1 Exerts Antihantaviral Effects by Acting as Positive Feedback for RIG-I Signaling

**DOI:** 10.1128/JVI.02250-16

**Published:** 2017-04-13

**Authors:** Hongwei Ma, Peijun Han, Wei Ye, Hesong Chen, Xuyang Zheng, Linfeng Cheng, Liang Zhang, Lan Yu, Xing'an Wu, Zhikai Xu, Yingfeng Lei, Fanglin Zhang

**Affiliations:** a Department of Microbiology, Fourth Military Medical University, Xi'an, China; b Center of Infectious Diseases, Tangdu Hospital, Fourth Military Medical University, Xi'an, China; Hudson Institute of Medical Research

**Keywords:** long noncoding RNA, NEAT1, Hantaan virus, RIG-I, DDX60, beta interferon, SFPQ, interferons, innate immunity

## Abstract

Hantavirus infection, which causes zoonotic diseases with a high mortality rate in humans, has long been a global public health concern. Over the past decades, accumulating evidence suggests that long noncoding RNAs (lncRNAs) play key regulatory roles in innate immunity. However, the involvement of host lncRNAs in hantaviral control remains uncharacterized. In this study, we identified the lncRNA NEAT1 as a vital antiviral modulator. NEAT1 was dramatically upregulated after Hantaan virus (HTNV) infection, whereas its downregulation *in vitro* or *in vivo* delayed host innate immune responses and aggravated HTNV replication. Ectopic expression of NEAT1 enhanced beta interferon (IFN-β) production and suppressed HTNV infection. Further investigation suggested that NEAT1 served as positive feedback for RIG-I signaling. HTNV infection activated NEAT1 transcription through the RIG-I–IRF7 pathway, whereas NEAT1 removed the transcriptional inhibitory effects of the splicing factor proline- and glutamine-rich protein (SFPQ) by relocating SFPQ to paraspeckles, thus promoting the expression of RIG-I and DDX60. RIG-I and DDX60 had synergic effects on IFN production. Taken together, our findings demonstrate that NEAT1 modulates the innate immune response against HTNV infection, providing another layer of information about the role of lncRNAs in controlling viral infections.

**IMPORTANCE** Hantaviruses have attracted worldwide attention as archetypal emerging pathogens. Recently, increasing evidence has highlighted long noncoding RNAs (lncRNAs) as key regulators of innate immunity; however, their roles in hantavirus infection remain unknown. In the present work, a new unexplored function of lncRNA NEAT1 in controlling HTNV replication was found. NEAT1 promoted interferon (IFN) responses by acting as positive feedback for RIG-I signaling. This lncRNA was induced by HTNV through the RIG-I–IRF7 pathway in a time- and dose-dependent manner and promoted HTNV-induced IFN production by facilitating RIG-I and DDX60 expression. Intriguingly, NEAT1 relocated SFPQ and formed paraspeckles after HTNV infection, which might reverse inhibitive effects of SFPQ on the transcription of RIG-I and DDX60. To the best of our knowledge, this is the first study to address the regulatory role of the lncRNA NEAT1 in host innate immunity after HTNV infection. In summary, our findings provide additional insights regarding the role of lncRNAs in controlling viral infections.

## INTRODUCTION

Hantaviruses belong to the family Bunyaviridae and genus Hantavirus, and they have attracted worldwide attention as archetypal emerging pathogens (1). Hantaviruses contain a tripartite negative-sense single-stranded RNA (ssRNA) genome, including small (S), medium (M), and large (L) genes, which encode nucleocapsid protein (NP), glycoprotein (GP), and viral RNA-dependent polymerase protein (RdRp), respectively. Humans become infected by inhaling contaminated aerosols or by coming into contact with rodent excreta, and they develop two severe acute diseases, namely, hemorrhagic fever with renal syndrome (HFRS) and hantavirus pulmonary syndrome (HPS) (2). Hantavirus infection affects up to 100,000 to 200,000 humans annually, with fulminant HFRS cases most represented in China (3). Chinese HFRS cases, mainly caused by Hantaan virus (HTNV) infection, account for approximately 90% of all global cases, with a mortality rate ranging from 0.1 to 15% (4). Since there is neither an effective therapeutic nor FDA-licensed vaccine, further understanding of host immune responses against hantaviral infection is of great significance for global public health and safety.

**FIG 1:**
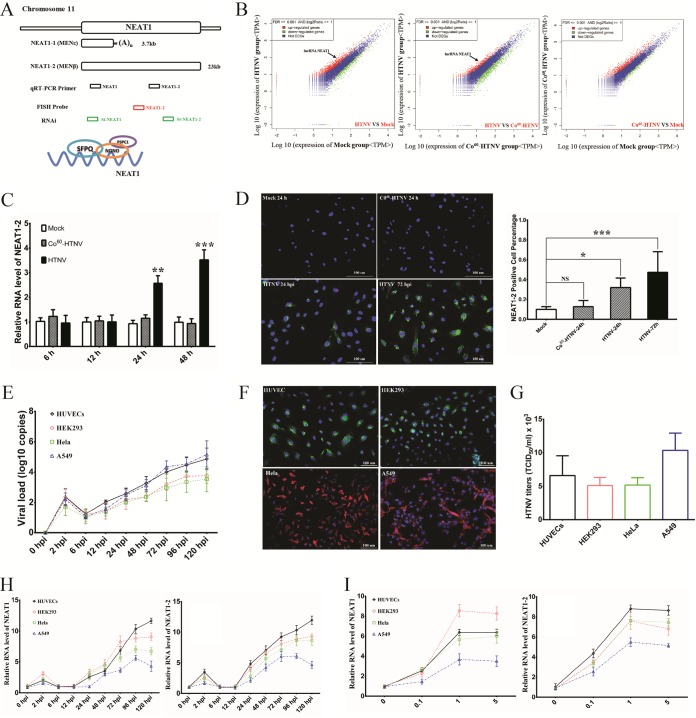
NEAT1 expression is induced by HTNV in a time- and dose-dependent manner. (A) The design for detecting and disrupting NEAT1 transcription, as well as the paraspeckle entities, is shown schematically. (B) RNA isolated from HUVECs, which were either mock infected or treated with live or ^60^Co-inactivated HTNV at an MOI of 1 for 24 h, was sequenced on an Illumina platform. More than 3.4 million reads per sample were obtained. Sequence analysis identified lncRNA NEAT1 as a significantly upregulated transcript after HTNV infection (indicated by arrows). (C) HUVECs were either mock infected or infected with HTNV or ^60^Co-inactivated HTNV, followed by qRT-PCR at 6, 12, 24, or 48 h to measure NEAT1-2. Values are means ± standard deviation (SD) (*n* = 5; *, *P* < 0.01; **, *P* < 0.001; ***, *P* < 0.0001; Student's *t* test, compared with the ^60^Co-inactivated HTNV group). (D) HUVECs were either mock infected or infected with HTNV or ^60^Co-inactivated HTNV, followed by FISH staining and immunostaining (left). NEAT1 (red), NP (green), and nuclei stained with DAPI (blue). The NEAT1-2-positive cell percentage was calculated based on four randomized fields in each group and analyzed using the Student *t* test (right). Values are means ± SD (*n* = 5; *, *P* < 0.01; **, *P* < 0.001; ***, *P* < 0.0001; compared with the mock group). (E) The expression levels of HTNV S segment in HUVECs or HEK293, HeLa, or A549 cells by qRT-PCR at 0, 2, 6, 12, 24, 48, 72, 96, and 120 hpi (infection MOI = 1, *n* = 5; replicates shown in the following data are biological). (F) Different cell lines were infected with HTNV and followed by immunostaining at 48 hpi. NP (shown as green in HUVECs and HEK293 cells and as red in HeLa and A549 cells) and nuclei stained with DAPI (blue). (G) The propagated HTNV in different cells was acquired at 72 hpi, and viral titers were detected by TCID_50_ with ELISA (*n* = 3). (H) The expression levels of NEAT1 were assessed in HUVECs or HEK293, HeLa, or A549 cells by qRT-PCR at different infection time intervals (MOI = 1, *n* = 5). (I) The expression levels of NEAT1 were assessed in HUVECs or HEK293, HeLa, or A549 cells by qRT-PCR at different infection MOIs (72 hpi, *n* = 5). At least three independent experiments were performed with similar results.

The innate immune system, characterized by interferon (IFN) responses and immunocyte activation, provides the initial defense against viral invasions. Cellular pathogen recognition receptors (PRRs), including Toll-like receptors (TLRs) and RIG-I like receptors (RLRs), can detect distinct pathogen-associated molecular patterns (PAMPs) and trigger the expression of IFNs and cytokines. RIG-I has been shown to recognize hantaviral invasion, but its regulatory process remains unclear (5). Long noncoding RNAs (lncRNAs) have emerged as important modulators of gene expression. lncRNA nuclear paraspeckle assembly transcript 1 (NEAT1) is an essential architectural constituent of paraspeckles in the mammalian nucleus, interacting with Drosophila DBHS RNA-binding proteins such as the splicing factor proline- and glutamine-rich protein (SFPQ) and the non-POU domain-containing, octamer-binding protein (NONO/p54) (6). To date, two isoform transcripts of the NEAT1 gene have been identified, namely, the 3.7-kb NEAT1-1 (MENε) and the 23-kb NEAT1-2 (MENβ) (Fig. 1A). A large amount of research has shown that NEAT1 is associated with oncogenesis and tumor progression (7–9), promoting cancer formation in mice by dampening oncogene-dependent activation of p53 (10). Nevertheless, studies assessing the function of NEAT1 in viral infections are scarce.

Here, the human umbilical vein endothelial cell (HUVEC) transcriptome was analyzed after HTNV infection by digital gene expression (DGE) profiling, and lncRNA NEAT1 was found to be remarkably upregulated by viral infection. Silencing NEAT1 *in vitro* or *in vivo* suppressed host immune responses and aggravated HTNV infection, whereas NEAT1 overexpression *in vitro* enhanced beta interferon (IFN-β) production and inhibited HTNV replication. Further investigation showed that NEAT1 promoted RIG-I and DDX60 expression by relocating SFPQ and removing the transcriptional inhibitory effects of SFPQ, which are critical for IFN responses against HTNV infection. We also found that RIG-I signaling, rather than TLR3 and TLR4, accounted for the elevation of HTNV-induced NEAT1. Taken together, our findings provide novel insights into the lncRNA-mediated regulatory mechanism of host innate defense against HTNV infection.

## RESULTS

### NEAT1 transcription is activated by HTNV in a time- and dose-dependent manner.

To explore the potential role of long noncoding RNAs in host innate immune responses, DGE analysis of HUVECs for whole-genome profiling was performed at 24 h post-HTNV infection. As shown in Fig. 1B, the NEAT1 level in the HTNV group was higher than that in the mock group (*P* = 6.86 × 10^−13^, false discovery rate [FDR] = 9.75 × 10^−12^) or the ^60^Co-inactivated HTNV group (*P* = 1.75 × 10^−14^, FDR = 3.10 × 10^−13^); however, the difference between the ^60^Co-inactivated HTNV group and the mock group was not significant (*P* = 0.21034, FDR = 0.58211). To confirm the profiling results, two primer pairs from the published literature (11), one recognizing both NEAT1-1 and NEAT1-2 and the other specific for NEAT1-2 (Fig. 1A), were applied to quantify NEAT1 RNA isoforms by quantitative real-time PCR (qRT-PCR). It has been reported that NEAT1-2 rather than NEAT1-1 plays a key regulatory role in paraspeckle formation (11), and we also found that elevated NEAT1 levels depend on live HTNV infection rather than ^60^Co-inactivated HTNV stimulation (Fig. 1C). Fluorescence *in situ* hybridization (FISH) with probes specific for NEAT1-2 was performed with HUVECs, and the results confirmed increased NEAT1-2 expression and the aggregation of NEAT1-2 in the nucleus at 24 and 48 h postinfection (hpi) (Fig. 1D).

To further investigate whether NEAT1 expression was altered in other cell lines, HEK293, HeLa, and A549 cells were used. All these cells could be infected by HTNV (Fig. 1E and F) and generated hantavirus progeny (Fig. 1G). Similar to the data obtained from HUVECs, NEAT1 was indeed upregulated by HTNV at a multiplicity of infection (MOI) of 1 beginning at 24 hpi in HUVECs and A549, HEK293, and HeLa cells, and the increasing tendency occurred in a time-dependent manner (Fig. 1H). Of note, the NEAT1 elevation at 2 hpi might have been unrelated to the virus but resulted in cellular stress responses. Besides, NEAT1 expression increased from an MOI of 0.1 to 1, indicating that the elevation occurred in a viral dose-dependent manner (Fig. 1I).

### NEAT1-2 and not NEAT1-1 suppresses HTNV replication in HUVECs.

**FIG 2:**
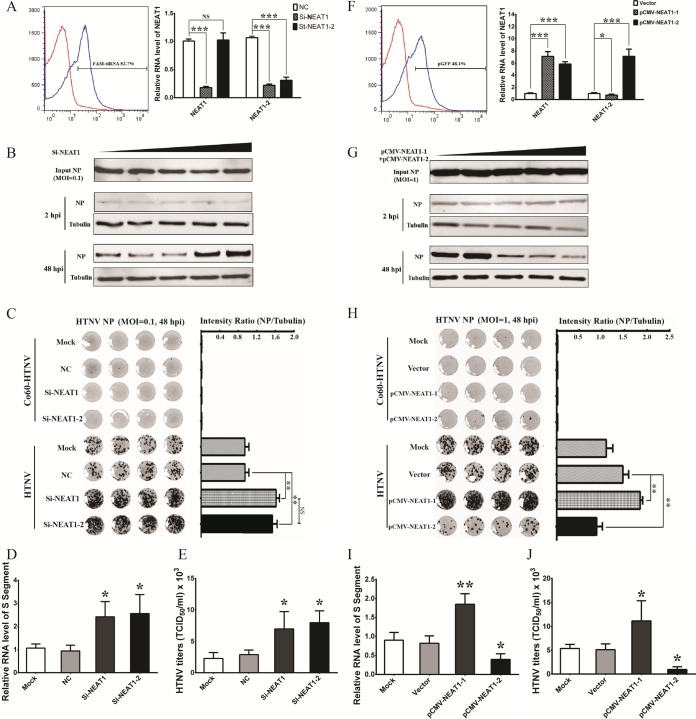
NEAT1-2 and not NEAT1-1 suppresses HTNV replication in HUVECs. (A) Left, HUVECs in six-well plates were transfected with nontarget control (NC) sequences or FAM (6-carboxyfluorescein)-siRNA (3 μg). Twenty-four hours after transfection, the cells expressing FAM were calculated by flow cytometry. Right, HUVECs in six-well plates were transfected with NC sequences, si-NEAT1, or the stealth siRNA NEAT1-2 (st-NEAT1-2) (3 μg). Twenty-four hours after transfection, the cells were infected with HTNV at an MOI of 1. At 48 hpi, the NEAT1 expression levels were measured by qRT-PCR. Values are means ± SD (*n* = 3; *, *P* < 0.01; **, *P* < 0.001; ***, *P* < 0.0001; Student's *t* test, compared with the NC group). NS, nonsignificant. (B) HUVECs in six-well plates were transfected with NC sequences (the amount of Si-NEAT1-2 is considered 0 μg) or increasing amounts of si-NEAT1 (0.1, 0.5, 1, and 3 μg). Twenty-four hours after transfection, the cells were infected with HTNV at an MOI of 0.1 for 48 h. The expression of HTNV NP was measured by Western blotting. (C) HUVECs were treated as described for panel A, right, but at an MOI of 0.1. In-cell Western (ICW) analysis for HTNV NP was performed at 48 hpi. The ICW for HTNV NP staining is shown on the left, while the relative intensity of fluorescence (NP/β-actin) was analyzed using Student's *t* test. (*n* = 4; *, *P* < 0.01; **, *P* < 0.001; Student's *t* test, compared with the NC group). (D) HUVECs were treated as described for panel A, right, but at an MOI of 0.1. The expression of HTNV S segment was measured by qRT-PCR. Values are means ± SD (*n* = 3; *, *P* < 0.01; Student's *t* test, compared with the NC group). (E) HUVECs were treated as described for panel A, right, but at an MOI of 0.1. The propagated HTNV was acquired at 72 hpi, and viral titers were detected by TCID_50_ with ELISA in Vero E6 cells. Values are means ± SD (*n* = 3; *, *P* < 0.01; Student's *t* test, compared with the NC group). (F) Left, HUVECs in six-well plates were transfected with vectors or pGFP (3 μg). Twenty-four hours after transfection, the cells expressing green fluorescent protein (GFP) were calculated by flow cytometry. Right, HUVECs in six-well plates were transfected with control plasmids (vector), pCMV-NEAT1-1, or pCMV-NEAT1-2 (3 μg). Twenty-four hours after transfection, the cells were infected with HTNV at an MOI of 1. At 48 hpi, the NEAT1 expression levels were measured by qRT-PCR. Values are means ± SD (*n* = 3; *, *P* < 0.01; **, *P* < 0.001; ***, *P* < 0.0001; Student's *t* test, compared with the vector group). (G) HUVECs in six-well plates were transfected with control plasmids (vector, the amount of pCMV-NEAT1-1 plus pCMV-NEAT1-2 is considered 0 μg) or increasing amounts of pCMV-NEAT1-1 plus pCMV-NEAT1-2 (0.05 + 0.05 μg, 0.25 + 0.25 μg, 0.5 + 0.5 μg, 1.5 + 1.5 μg, respectively). Twenty-four hours after transfection, the cells were infected with HTNV at an MOI of 1 for 48 h. The expression of HTNV NP was measured by Western blotting. (H) HUVECs were treated as described for panel F, right. In-Cell Western (ICW) analysis for HTNV NP was performed at 48 hpi. The ICW for HTNV NP staining is shown on the left, while the relative intensity of fluorescence (NP/β-actin) was analyzed using Student's *t* test. (*n* = 4; *, *P* < 0.01; **, *P* < 0.001; Student's *t* test, compared with the vector group). (H) HUVECs were treated as described for panel F, right. The expression of HTNV S segment was measured by qRT-PCR. Values are means ± SD (*n* = 3; *, *P* < 0.01; **, *P* < 0.001; Student's *t* test, compared with the vector group). (I) HUVECs were treated as described for panel F, right. The propagated HTNV was acquired at 72 hpi, and viral titers were detected by TCID_50_ with ELISA in Vero E6 cells. Values are means ± SD (*n* = 3; *, *P* < 0.01; Student's *t* test, compared with the vector group).

The above-described data showed that HTNV infection increased NEAT1, and we wondered how NEAT1 could reciprocally influence HTNV replication. The small interfering RNA (siRNA) transfection efficiency in HUVECs was confirmed by flow cytometry, and NEAT1 expression was significantly decreased, as assessed by qRT-PCR after RNA interference (RNAi) (Fig. 2A). Of note, si-NEAT1 targets both NEAT1-1 and NEAT1-2, whereas the stealth siRNA NEAT1-2 (st-NEAT1-2) is specific for NEAT1-2. Compared with the cells transfected with control siRNA (negative control [NC]), HUVECs with si-NEAT1 could dramatically promote HTNV NP production, and NP expression seemed to be related to the amount of applied si-NEAT1 (Fig. 2B). Intriguingly, depletion of NEAT1-2 alone could mimic the antiviral effects of simultaneous NEAT1-1 and NEAT1-2 silencing (Fig. 2C), indicating that NEAT1-2 was critical for the antiviral responses. Consistent with those data, the expressions of HTNV mRNA of S segment (Fig. 2D) and HTNV titers (Fig. 2E) were increased after NEAT1 silencing.

On the other hand, plasmids, of which pCMV-NEAT1-1 is transcribed into the 3.7-kb NEAT1-1 (MENε) and pCMV-NEAT1-2 is transcribed into the 2- to 3-kb NEAT1-2 (MENβ), were applied to directly investigate the role of NEAT1 in HTNV infection (Fig. 2F). Surprisingly, we found NEAT1-1 overexpression restricted NEAT1-2 transcription (Fig. 2F). Overexpression of NEAT1 with both pCMV-NEAT1-1 and pCMV-NEAT1-2 could conspicuously repress HTNV NP expression, and NP expression seemed to be associated with the transfected plasmids (Fig. 2G). Furthermore, overexpression of NEAT1-2 instead of NEAT1-1 could efficiently suppress HTNV replication (Fig. 2H). NEAT1-1 upregulation even aggravated HTNV infection (Fig. 2H), which may be the result of downregulation of NEAT1-2. Consistently, through analysis of viral load detected by qRT-PCR and the 50% tissue culture infective dose (TCID_50_) test by ELISA, we found that expression of HTNV-specific mRNA (Fig. 2I) and HTNV titers (Fig. 2J) were limited in HUVECs in which NEAT1-2 was ectopically expressed in comparison to those transfected with control vector or pCMV-NEAT1-1. These data further showed that NEAT1-2 and not NEAT1-1 suppresses HTNV replication in HUVECs.

### Alteration of NEAT1-2 affects HTNV-induced IFN expression in HUVECs.

**FIG 3:**
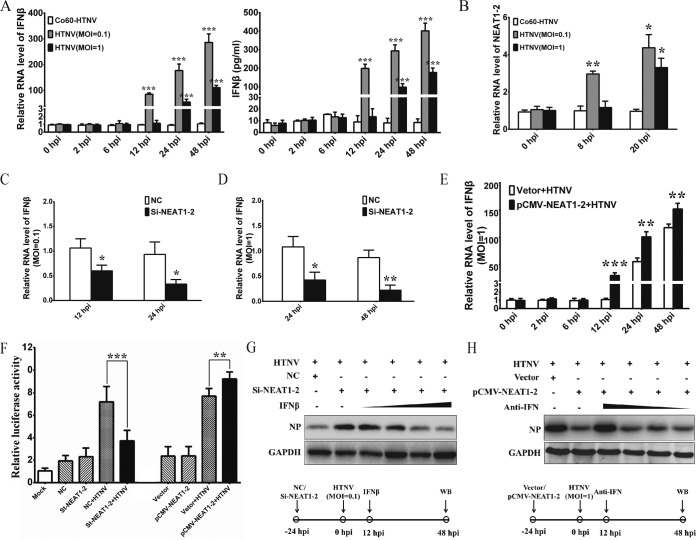
Alteration of NEAT1 affects HTNV-induced IFN expression in HUVECs. (A) HUVECs were infected with either ^60^Co-inactivated HTNV or HTNV at an MOI of 0.1 or 1. IFN-β expression at different time points was assessed by qRT-PCR (left) and ELISA (right). Values are means ± SD (*n* = 3; *, *P* < 0.01; Student's *t* test, compared with the ^60^Co-inactivated HTNV group). (B) HUVECs were infected with either ^60^Co-inactivated HTNV or HTNV at an MOI of 0.1 or 1. The NEAT1-2 expression at different time points was assessed by qRT-PCR. Values are means ± SD (*n* = 3; *, *P* < 0.01; Student's *t* test, compared with the ^60^Co-inactivated HTNV group). (C and D) HUVECs were transfected with NC or si-NEAT1-2 and, 24 h later, were infected with HTNV at an MOI of 0.1 (C) or 1 (D). IFN-β expression at different time points was assessed by qRT-PCR. Values are means ± SD (*n* = 3; *, *P* < 0.01; Student's *t* test, compared with the NC group). (E) HUVECs were transfected with control plasmids (vector) or pCMV-NEAT1-2 and, 24 h later, were infected with HTNV at an MOI of 1. IFN-β expression at different time points was assessed by qRT-PCR. Values are means ± SD (*n* = 3; *, *P* < 0.01; Student's *t* test, compared with the vector plus HTNV group). (F) Detection for luciferase reporter activities driven by the IFN-β promoter was done in siRNA or plasmid-pretransfected HUVECs without HTNV infection or with HTNV infection for 48 h (MOI = 5). Values are means ± SD (*n* = 5; *, *P* < 0.01; **, *P* < 0.001; Student's *t* test). (G) HUVECs were transfected with NC or si-NEAT1-2 and, 24 h later, were infected with HTNV at an MOI of 0.1. Increasing amounts of IFN-β were added starting at 12 hpi. The HTNV NP production was measured by Western blotting at 48 hpi. (H) HUVECs were transfected with control plasmids (vector) or pCMV-NEAT1-2 and, 24 h later, were infected with HTNV at an MOI of 1. Decreasing amounts of polyclonal sheep neutralizing antibodies against IFN-α and IFN-β (anti-IFN) were added starting at 12 hpi. The HTNV NP production was measured by Western blotting at 48 hpi.

IFN-β production or pretreatment at an early infection stage plays an important role in limiting HTNV infection, while IFN-β treatment after 24 hpi exerts little antiviral effect (12, 13). It has been reported that the GnT of hantaviruses suppressed IFN-β expression of host cells at an early stage of infection (14). Here, we also found that HUVECs could not efficiently produce IFN-β until 12 hpi at an MOI of 0.1 or until 24 hpi at an MOI of 1 (Fig. 3A), which indicated that high doses of HTNV could hamper prompt IFN responses. Notably, enhanced NEAT1-2 transcription appeared at 8 hpi at an MOI of 0.1 or at 20 hpi at an MOI of 1 (Fig. 3B), suggesting that NEAT1-2 expression increased just before IFN production. We found that expression of endogenous IFN-β mRNA was much lower in cells transfected with si-NEAT1-2 at MOIs of both 0.1 (Fig. 3C) and 1 (Fig. 3D) than in those transfected with control siRNA (NC). In contrast, overexpression of NEAT1 in HUVECs increased IFN-β expression after HTNV infection (MOI = 1) at 24 and 48 hpi (Fig. 3E). More importantly, HUVECs transfected with pCMV-NEAT1-2 conspicuously increased IFN-β gene expression compared with those cells with vector plasmids at 12 hpi (MOI = 1), demonstrating that NEAT1-2 overexpression accelerated robust IFN responses in host cells against HTNV infection. With a dual luciferase reporter system maintaining IFN-β promoters, we found NEAT1-2 silencing or overexpression could inhibit or increase the promoter activity of the IFN-β gene after HTNV infection, respectively, whereas silencing NEAT1-2 or ectopically expressing NEAT1-2 without HTNV infection could not inhibit or enhance IFN-β expression (Fig. 3F). These results showed that NEAT1-2 regulated HTNV-induced IFN-β expression.

To explore whether the antihantavirus effects of NEAT1 were caused by IFN-β alteration, a series of compensatory experiments was designed. In NEAT1-2 knockdown HUVECs, the addition of IFN-β at 12 hpi could efficiently block HTNV NP production (MOI = 0.1), and such phenomena were also determined by the amount of applied IFN-β (Fig. 3G). In addition, in cells with high NEAT1-2 expression, treatment with neutralizing antibodies (NAbs) of IFN-α and IFN-β could counteract the antiviral effects of NEAT1-2 (MOI = 1), and the compensatory effects were dependent on the magnitude of the NAbs. Together these results demonstrated that NEAT1-2 especially enhanced the host antihantaviral innate immune responses by regulating IFN-β signaling.

### RIG-I and DDX60 regulated by NEAT1-2 facilitate HTNV-induced IFN-β production.

**FIG 4:**
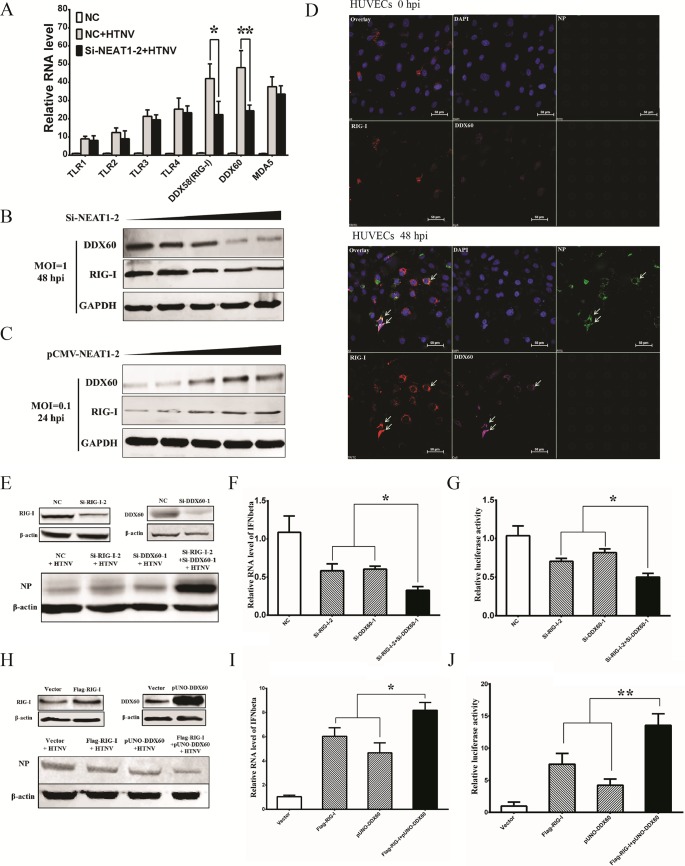
RIG-I and DDX60 regulated by NEAT1 facilitates HTNV-induced IFN-β production. (A) HUVECs transfected with NC or si-NEAT1-2 were mock infected or infected with HTNV at an MOI of 1, and expression levels of TLR1, TLR2, TLR3, TLR4, RIG-I, DDX60, and MDA5 were measured by qRT-PCR at 48 hpi. Values are means ± SD (*n* = 3; *, *P* < 0.01; **, *P* < 0.001; Student's *t* test). (B and C) HUVECs with increasing amounts of si-NEAT1-2 (B) or pCMV-NEAT1-2 (C) were infected with HTNV at an MOI of 1, and the RIG-I and DDX60 production levels were measured by Western blotting at selected time points. (D) HUVECs infected with HTNV at an MOI of 1 were subjected to visualization of RIG-I (red) and DDX60 (green) by immunostaining at 48 hpi; counterstaining was conducted with DAPI (blue). (E and F) HEK293 cells were transfected with NC sequences, si-RIG-I-2, si-DDX60-1, or both si-RIG-I-2 and si-DDX60-1. Twenty-four hours after transfection, the cells were infected with HTNV at an MOI of 1. The knockdown efficiencies of RIG-I or DDX60 (E, upper panels), as well as HTNV NP expression (E, lower panels), were assessed at 48 hpi by Western blotting. qRT-PCR was performed to detect the expression of IFN-β. (F) Values are means ± SD (*n* = 5; *, *P* < 0.01; **, *P* < 0.001; one-way ANOVA). (G) A luciferase gene harboring the IFN-β promoter was transfected with si-RIG-I-2 and/or si-DDX60-1 in HEK293 cells, and after 24 h, the HEK293 cells were infected with HTNV at an MOI of 5. At 48 hpi, the relative luciferase activity for IFN-β was compared with that in cells that received a mock transfection, as determined by the luminescence intensity. Values are means ± SD (*n* = 5; *, *P* < 0.01; **, *P* < 0.001; one-way ANOVA). (H and I) HEK293 cells were transfected with vector plasmids, Flag-RIG-I, pUNO-DDX60, or both Flag-RIG-I and pUNO-DDX60. Twenty-four hours after transfection, the cells were infected with HTNV at an MOI of 1. The overexpression efficiencies of RIG-I or DDX60 (H, upper panels), as well as HTNV NP expression (H, bottom panels), were assessed at 48 hpi by Western blotting. Additionally, qRT-PCR was performed to detect the expression of IFN-β (I) in HEK293 cells. Values are means ± SD (*n* = 5; *, *P* < 0.01; **, *P* < 0.001; one-way ANOVA). (J) A luciferase gene harboring the IFN-β promoter was transfected with Flag-RIG-I and/or pUNO-DDX60 in HEK293 cells. Twenty-four hours later, the cells were infected with HTNV at an MOI of 5. At 48 hpi, the relative luciferase activity for IFN-β was compared with values for mock-transfected cells, as determined by the luminescence intensity. Values are means ± SD (*n* = 5; *, *P* < 0.01; **, *P* < 0.001; one-way ANOVA). The experiments were performed independently at least three times with similar results.

PRRs maintain a vital role in the promotion of IFN responses, and we conjectured that NEAT1 might amplify IFN responses by modulating these molecules. TLR3, TLR4, and RIG-I have been shown to recognize HTNV infection (5, 15, 16). DDX60 was recently reported as an important activator of RIG-I, but the antiviral effects of DDX60 remain a subject of debate (17, 18), Here, we found that multiple Toll-like receptors like TLR1, TLR2, TLR3, and TLR4, as well as MDA5, were increased after HTNV infection, but none of them were influenced by silencing NEAT1-2 (Fig. 4A). The upregulated RIG-I and DDX60 were blocked in the cells with low NEAT1-2 expression after HTNV infection (Fig. 4A). HUVECs with declining NEAT1-2 expression showed gradually decreasing expression of RIG-I and DDX60 (Fig. 4B), and increasing NEAT1-2 transcription was found to activate RIG-I and DDX60 production accordingly (Fig. 4C). These data indicated that NEAT1-2 could positively modulate RIG-I and DDX60 expression, while the role of RIG-I and DDX60 upon HTNV infection is obscure.

We then found that RIG-I and DDX60 colocalized after HTNV infection (Fig. 4D), implying that RIG-I and DDX60 might collaborate with each other to exert antiviral effects. To verify the antiviral role of RIG-I and DDX60, we designed a series of siRNAs targeting RIG-I and DDX60, and we selected the si-RIG-I-2 and siRNA-DDX60-1 with the highest knockdown efficiency by qRT-PCR in HUVECs (data not shown). Simultaneously knocking down RIG-I and DDX60 significantly promoted HTNV NP expression (Fig. 4E), and knockdown of both of them could greatly affect IFN-β expression (Fig. 4F and G). Ectopic expression of either RIG-I or DDX60 inhibited viral replication, whereas overexpression of both resulted in superior antiviral effects (Fig. 4H), indicating that efficient anti-HTNV responses might depend on the interactive effects of DDX60 and RIG-I. More importantly, RIG-I or/and DDX60 overexpression enhanced HTNV-induced IFN-β expression, and they had synergistic effects on IFN-β production (Fig. 4I and J). Consequently, NEAT1 might regulate IFN-β production by upregulating RIG-I and DDX60, and thus we were interested in how NEAT1 regulated RIG-I and DDX60 expression.

### SFPQ, which is relocated by NEAT1 HTNV infection, regulates the expression of RIG-I and DDX60.

**FIG 5:**
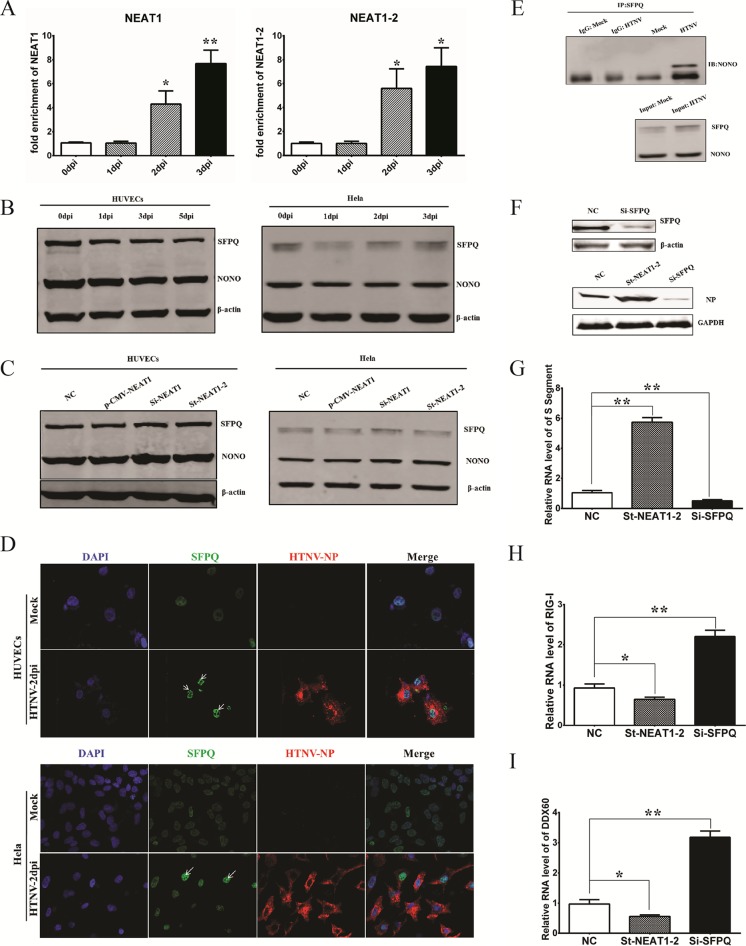
SFPQ that is relocated by NEAT1 HTNV infection regulates the expression of RIG-I and DDX60. (A) HUVECs were infected with HTNV at an MOI of 1, and then, to detect the interaction of NEAT1 with SFPQ, RIP was performed at different time points. Values are means ± SD (*, *P* < 0.01; **, *P* < 0.001; one-way ANOVA). (B and C) The levels of the paraspeckle proteins SFPQ and NONO in HUVECs or HeLa cells were assessed by Western blotting at the indicated time points after HTNV infection (B) or posttransfection with plasmids or siRNA (C), using β-actin as a loading control. (D) The SFPQ location before and after HTNV infection, both in HUVECs and in HeLa cells, was detected by immunostaining. (E) The enhanced interplay between SFPQ and NONO after HTNV infection in HEK293 cells was verified by co-IP. (F, G, H, and I) HEK293 cells were transfected with NC, st-NEAT1-2, or si-SFPQ and, 24 h later, were infected with HTNV at an MOI of 1. At 2 dpi, the silencing efficiency of si-SFPQ was assessed in HeLa cells by Western blotting (F, upper panels); HTNV NP was measured by Western blotting at 2 dpi (F, bottom panels). The transcription of HTNV S segment (G) (*n* = 3), RIG-I (H) (*n* = 3), and DDX60 (I) (*n* = 3) was detected by qRT-PCR at 2 dpi. Values are means ± SD (*, *P* < 0.01; **, *P* < 0.001; Student's *t* test). The experiments were performed independently at least three times with similar results.

NEAT1 was found to interact with SFPQ by RNA immunoprecipitation (RIP) after HTNV infection (Fig. 5A), indicating that modulatory effects of NEAT1 might be involved in SFPQ. Interestingly, the protein level of SFPQ, as well as another paraspeckle-forming constituent, NONO, remained unchanged after HTNV infection (Fig. 5B) or after NEAT1 overexpression and knockdown (Fig. 5C). However, SFPQ became centralized rather than diffuse in the nucleus after HTNV infection (Fig. 5D). The enhanced interaction of SFPQ and NONO indicated excess formation of paraspeckles in the nucleus (Fig. 5E) and relocalization of SFPQ. SFPQ knockdown could inhibit HTNV replication (Fig. 5F and G), which might have been related to the increase in RIG-I (Fig. 5H) and DDX60 (Fig. 5I). SFPQ has been suggested to bind to the promoter region of RIG-I and DDX60 (11), thus preventing the expression of RIG-I and DDX60. Taken together, the above results suggested that NEAT1 might relocate SFPQ from the potential promoter region to paraspeckles in the nucleus and remove the transcriptional inhibitory effects of SFPQ, thus facilitating the expression of RIG-I and DDX60.

### RIG-I signaling is crucial for NEAT1 expression after HTNV infection.

**FIG 6:**
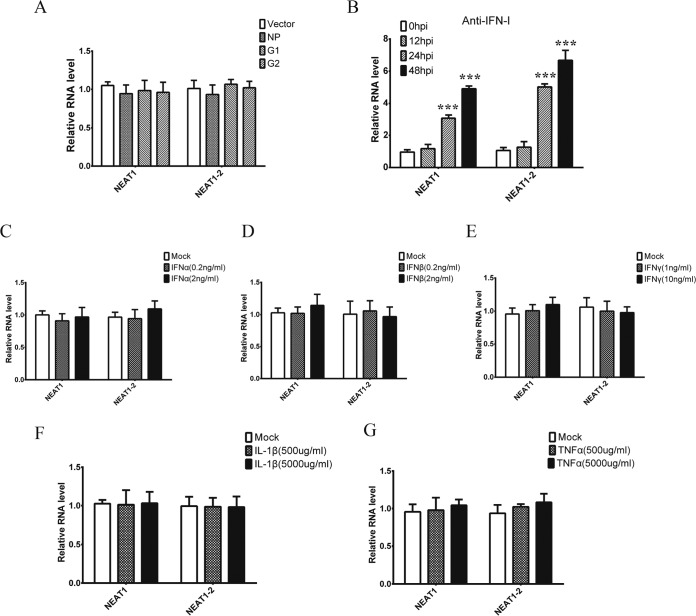
NEAT1 upregulation is dependent on live viral replication. (A) HEK293 cells were transfected with pcDNA3.1 (vector), pcDNA3.1-NP, pcDNA3.1-G1, or pcDNA3.1-G2 for 48 h, and NEAT1 levels were tested by qRT-PCR. Values are means ± SD (*, *P* < 0.01; **, *P* < 0.001; one-way ANOVA). (B) HUVECs were mock infected or HTNV infected with polyclonal sheep antibodies against IFN-α and IFN-β (1:100 dilution; anti-IFN-I), and the cells were collected for NEAT1 detection by qRT-PCR at different times postinfection. Values are means ± SD (*, *P* < 0.01; **, *P* < 0.001; Student's *t* test, compared with results at 0 dpi). (C, D, and E) HUVECs were stimulated with IFN-α (C), IFN-β (D), or IFN-γ (E) at various doses for 12 h; qRT-PCR was then performed to assess NEAT1 expression levels. Values are means ± SD (*, *P* < 0.01; **, *P* < 0.001; one-way ANOVA). (F and G) HUVECs were stimulated with IL-1β (F) or TNF-α (G) at various doses for 12 h, and then qRT-PCR was performed to assess NEAT1 expression levels. Values are means ± SD (*, *P* < 0.01; **, *P* < 0.001; one-way ANOVA). The experiments were performed independently at least three times with similar results.

Elevated NEAT1 exerts antiviral effects by modulating the innate immune response, yet it is unclear how HTNV triggers NEAT1 transcription. Interestingly, overexpression of the S or M segment of HTNV in HEK293 cells failed to induce NEAT1 expression, suggesting that NEAT1 transcription was closely related to live viral replication (Fig. 6A). Of note, the upregulation of NEAT1 by HTNV could not be reversed by applying IFN-I neutralizing antibodies (Fig. 6B). Meanwhile, NEAT1 expression could not be induced by stimulation with different types of IFNs (Fig. 6C, D, and E) or cytokines (Fig. 6F and G).

**FIG 7:**
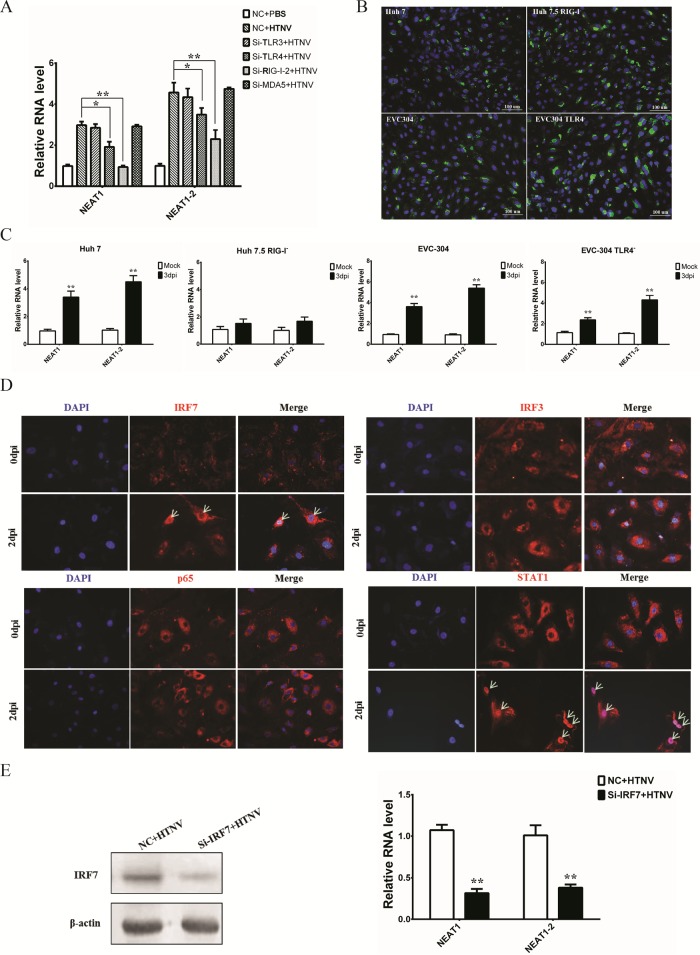
RIG-I signaling is crucial for NEAT1 expression after HTNV infection. (A) After TLR3, TLR4, RIG-I, and MDA5 silencing, HUVECs were infected with HTNV at an MOI of 1, and NEAT1 levels were measured by qRT-PCR at 2 dpi (*n* = 3). Values are means ± SD (*, *P* < 0.01; **, *P* < 0.001; Student's *t* test, compared with the NC+HTNV group). (B) Huh7, Huh7.5, EVC-304, and EVC304 TLR4^−^ cells were infected with HTNV at an MOI of 1, followed by immunostaining at 48 hpi. NP (green) and nuclei stained with DAPI (blue) are shown. (C) NEAT1 levels in Huh7 and Huh7.5 (RIG-I^−^) cells, as well as EVC-304 and EVC-304 TLR4^−^ cells, were assessed by qRT-PCR at 0 or 3 dpi (*n* = 3). Values are means ± SD (*, *P* < 0.01; **, *P* < 0.001). (D) Nuclear staining of IRF7 and STAT1, but not IRF3 or p65, was observed in HTNV-infected HUVECs. (E) NEAT1 transcription levels after HTNV infection were analyzed by qRT-PCR in NC-transfected or IRF7 silenced HUVECs (Right, *n* = 3). Values are means ± SD (*, *P* < 0.01; **, *P* < 0.001; Student's *t* test, compared with the NC+HTNV group). The experiments were performed independently at least three times with similar results.

We conjectured that NEAT1 expression was related to the activation of PRRs. By knocking down several PRRs, we found that the RIG-I and TLR4 pathways played important roles in HTNV-induced NEAT1 upregulation (Fig. 7A). Using RIG-I- and TLR4-deficient cell lines which could be well infected by HTNV (Fig. 7B), RIG-I was confirmed to be indispensable for NEAT1 induction after HTNV infection (Fig. 7C). Moreover, using STAT1 as a positive control, we found that the transcription factor IRF7, rather than IRF3 and p65, translocated into the nucleus in HTNV-infected HUVECs at 2 dpi (Fig. 7D). Furthermore, IRF7 knockdown blocked HTNV-induced NEAT1 upregulation (Fig. 7E). Therefore, HTNV caused transcriptional activation of the NEAT1 gene, probably via the RIG-I–IRF7 pathway.

### NEAT1 silencing has profound effects on innate immune responses after HTNV infection in mice.

**FIG 8:**
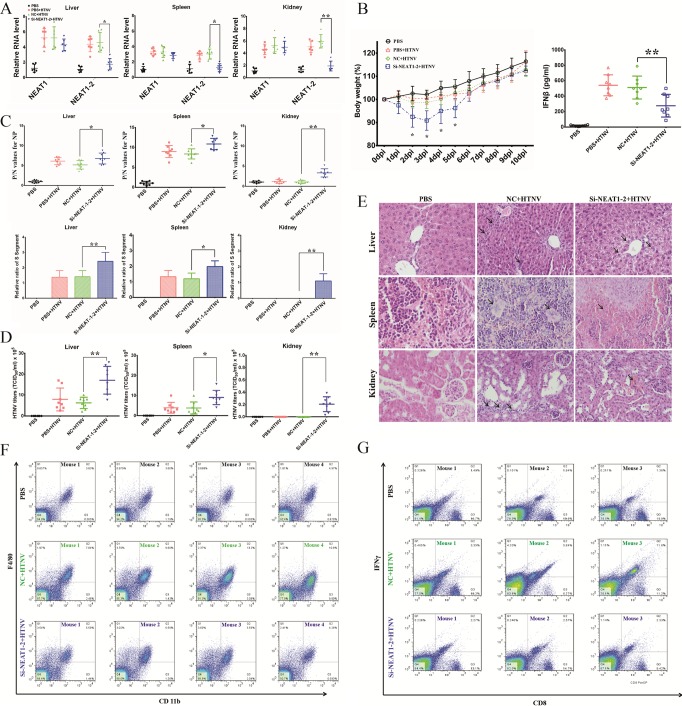
Silencing NEAT1 has profound effects on the innate immune response after HTNV infection in mice. To further determine the function of NEAT1 after HTNV infection *in vivo*, mice were injected intravenously with si-NEAT1-2 (1 μg/g) or nontarget control siRNA (NC) (1 μg/g); 1 day later, they were infected with HTNV (100 LD_50_) by intramuscular injection. (A) To maintain high knockdown efficiency, siRNAs were injected intravenously every other day. (A) The knockdown efficiency was assessed by qRT-PCR in kidney, liver, and spleen samples at 2 dpi (*n* = 6 in each group). (B) The effects of NEAT1 on HTNV virulence in mice were determined by body weight loss from 0 to 10 dpi (left panel, *n* = 10 in each group). The IFN-β in sera of different groups was measured by ELISA at 3dpi (right panel, *n* = 8 in each group). Values are means ± SD (*, *P* < 0.01; **, *P* < 0.001; Student's *t* test, compared with the NC+HTNV group). (C) Mice were sacrificed at 3 dpi, and livers, spleens, and kidneys were collected for ELISA detection of HTNV NP titers (upper panels, *n* = 8 in each group) and qRT-PCR to assess HTNV S segment levels (bottom panels, *n* = 8 in each group) at 3 dpi. Values are means ± SD (*, *P* < 0.01; **, *P* < 0.001; Student's *t* test, compared with the NC+HTNV group). (D) NEAT1 effects on HTNV infection kinetics at 3 dpi were determined by testing the HTNV titers in livers, spleens, and kidneys. Values are means ± SD (*n* = 8; *, *P* < 0.01; **, *P* < 0.001; Student's *t* test, compared with the NC+HTNV group). (E) Hematoxylin and eosin (H&E) staining for mouse liver, spleen, or kidney specimens was performed (3 dpi, *n* = 8 in each group). After HTNV infection, livers in the NC group showed inflammatory cell infiltration in certain regions, while those in the si-NEAT1-2 group showed slight acute viral hepatitis. Spleens in the NC group showed lymph node hyperplasia, while those in the si-NEAT1-2 group were severely congestive. Kidneys in the NC group also showed inflammatory cell infiltration, while those in the si-NEAT1-2 group had moderate interstitial congestion. (F) Macrophage infiltration in spleens was analyzed by detecting CD11b and F4/80 by flow cytometry at 3 dpi, and the results obtained for four mice in each group are presented. (G) CD3^+^ CD8^+^ IFN-γ^+^ T cells were analyzed by flow cytometry at 3 dpi, and the results obtained for three mice in each group are presented.

Although cell-based experiments revealed that NEAT1-2 is a crucial regulator of innate antihantaviral responses, its function *in vivo* has remained unclear. To address this question, we intravenously injected siRNAs targeting mouse NEAT1-2 at 1 day before HTNV infection. NEAT1-2 expression levels in the liver, kidney, and spleen were reduced at 2 dpi (Fig. 8A). Previous studies have shown that NEAT1 knockout does not affect physiological processes except *potentia generandi* in mice; hence, we assessed its role under pathological conditions. Body weight loss in NEAT1-2-depleted mice was observed from 2 dpi to 5 dpi, and the IFN production in serum was remarkably decreased in the NEAT1-2 silenced group than those in the NC group at 3 dpi (Fig. 8B). As expected, NEAT1-2 knockdown mice showed considerably higher HTNV NP levels in the liver, spleen, and kidney at 3 dpi (Fig. 8C). Moreover, the virus titers in related organs were higher in the NEAT1-2 silenced group than in the NC group (Fig. 8D). In addition, reduced inflammatory cell filtration but increased tissue injury was found in NEAT1-2 knockdown mice during the early stage of infection (Fig. 8E). Infiltration of macrophages in the spleen was attenuated (Fig. 8F), and the activation of macrophages was also suppressed (by flow cytometry; data not shown). Moreover, CD8^+^ IFN-γ^+^ T cells were reduced in the spleens of NEAT1-2 knockdown mice in comparison to those in the NC group at 3 dpi (Fig. 8G). Nevertheless, NEAT1-2 silencing had no effect on the production of neutralizing antibodies at 7 dpi (data not shown). The above-described findings indicated that NEAT1-2 depletion might influence multiple aspects of the innate immune response in HTNV-infected mice.

## DISCUSSION

Innate immunity is a phylogenetically ancient and conserved system that counteracts invading microbes, the regulatory mechanism of which is sophisticated and complex. Long noncoding RNAs, which were once considered dark materials in the mammalian genome, have been shown to exert vital modulatory effects on host innate immunity (19). In this report, we first demonstrated that NEAT1 was induced by HTNV through the RIG-I–IRF7 pathway and served as positive feedback for RIG-I signaling. Using DGE analysis, we observed upregulated NEAT1 and confirmed its alteration in different cell lines. To assess its effects on HTNV replication, NEAT1 was silenced both *in vitro* and *in vivo*, which resulted in increased HTNV infection and suppressed innate immune responses. Further analysis indicated that NEAT1 might interact with SFPQ and regulate DDX60 and RIG-I expression. By virtue of RNAi, the RIG-I–IRF7 pathway was confirmed to be necessary for HTNV-triggered NEAT1 elevation.

Recently, large-scale transcriptomic studies identified numerous noncoding transcripts in the mammalian genome, which were speculated to influence diverse biological processes. Among these noncoding RNAs (ncRNAs), long noncoding RNAs (lncRNAs) emerged as important regulators of gene expression and are closely related to the activation of the host innate immune system. TLR2 controls lncRNA-COX2 expression in a MyD88- and NF-κB-dependent manner, whereas lncRNA-COX either promotes interleukin 6 (IL-6) secretion or represses ISG15 and CCL5 expression (20). TLR2 activation or tumor necrosis factor alpha (TNF-α) stimulation induces transcription of the lncRNA THRIL, the downregulation of which impairs TNF-α and IL-6 secretion (21). TLR4 signaling in response to lipopolysaccharide (LPS) induces lncRNA IL-1β-eRNA and IL-1β-RBT46, the knockdown of which attenuates IL-1β and CXCL8 release (22). The lncRNA Lethe, triggered by TNF-α and IL-1β, acts as a negative feedback regulator of NF-κB signaling (23).

The roles of lncRNAs in host-virus interactions have been progressively unveiled. Various viruses, such as influenza virus (IAV), coronavirus, enterovirus, human immunodeficiency virus (HIV), hepatitis B virus (HBV), hepatitis C virus (HCV), Japanese encephalitis virus (JEV), and rabies virus, have been reported to activate the transcription of different lncRNAs in host cells (11, 24–26). Importantly, multiple lncRNAs have been shown to affect the IFN response in recent years and have gradually become hot spots in the field of antiviral research. NeST was shown to enhance IFN-γ production, controlling the susceptibility of mice to persistent Theiler's virus infection as well as resistance to Salmonella enterica serovar Typhimurium infection (27). Both CMPK2 and NRAV were identified as negative regulators of IFN immune reactions. CMPK2, induced by IFN-α or HCV infection, suppresses various ISGs, the knockdown of which dramatically blocks HCV replication (26). NRAV inhibits some critical ISGs, such as IFITM3 and Mx1, the depletion of which suppresses IAV replication both *in vitro* and *in vivo* (25). Numerous lncRNAs, including lnc-ISG15 and ISR2, respond to IFNs such as ISGs, although their actual function requires further investigation (28). Considering the poor evolutionary conservation but rapid divergence of lncRNAs, their functions may be highly species and virus specific. Though considerable progress has been achieved to demonstrate the antiviral effects of lncRNAs on model viruses, there are no published reports assessing the role of lncRNAs in hantaviral infection.

NEAT1 has been reported to interact with Drosophila DBHS RNA-binding proteins (e.g., SFPQ, NONO-p54nrb, and PSPC1), recruiting them to paraspeckles, a nuclear substructure found in all cultured and primary cells except embryonic stem cells (24). The versatile function of NEAT1 is rapidly progressing in multiple areas of biology. NEAT1 has been reported to be involved in the pathogenesis of multiple types of cancer (7–9). NEAT1 also participates in neurodegenerative diseases such as Huntington's disease (29) and seems to potentially contribute to the elevated production of a number of cytokines and chemokines in patients with systemic lupus erythematosus (SLE) (30). Furthermore, poly I·C can activate NEAT1 transcription through the TLR3 pathway, whereas NEAT1 positively regulates IL-8 transcription and potentially affects the expression of multiple ISGs after poly I·C stimulation (11). In addition, NEAT1 has been reported to suppress the export of Rev-dependent instability element (INS)-containing HIV-1 mRNAs from the nucleus to the cytoplasm, thus inhibiting HIV replication (24). However, the role of NEAT1 in hantaviral infection remains unclear.

In this report, NEAT1 has been identified as an important regulator of the host innate immune system against HTNV infection. Elevated NEAT1 promotes IFN secretion, most likely by enhancing RIG-I and DDX60 expression. DDX60, a DEXD/H box RNA helicase similar to Saccharomyces cerevisiae Ski2, is induced after viral infection (31). DDX60 recognizes viral RNA and activates endogenous RIG-I, thereby promoting the RIG-I signaling-related IFN response. However, the antiviral effects of DDX60 seem to vary among viruses (17). We found that NEAT1-regulated DDX60 was involved in IFN production in response to HTNV infection. In HTNV-infected cells, double-stranded RNA (dsRNA) could not be detected, and it is unclear how host PRRs, especially RIG-I, recognize HTNV invasion (5). Here, considering the interaction of RIG-I and DDX60 and the effect of DDX60 on IFN-β production, we hypothesize that DDX60 might mediate RIG-I signaling activation upon HTNV infection, which requires further investigation.

Of note, we applied multiple cell lines to explore the role of NEAT1 during HTNV infection. HTNV primarily targets vascular endothelial cells *in vivo* and contributes to the increased vascular permeability and coagulation disorders in HFRS; hence, HUVECs are the most common *in vitro* cell model to study host innate immunity against HTNV infection or viral pathogenesis (32). EVC-304 cells are also endovascular cells, whereas EVC-304 TLR4^−^ cells are TLR4-deleted cells, both of which have been used for HTNV infection related studies (15, 33). A549 cells were once used to isolate HTNV, and they were confirmed to be a mature model of infection (34–37). Additionally, Huh 7.0 and Huh 7.5 (RIG-I^−^) cells used in our study have been reported to be infected by HTNV by Lee et al. (5) and can be used as a cell model to study immune responses against HTNV replication (38, 39). Additionally, HEK293 (40) and HeLa (41) cells have been reported to be infected by HTNV. Using qRT-PCR, Western blotting, and immunofluorescence assays, we have also shown that both HEK293 and HeLa cells can be infected by HTNV. To study the molecular mechanism underlying the effect of NEAT1 on IFN expression and HTNV infection, it may be suitable to use HEK293 and HeLa cells as a cell model, especially under conditions in which HTNV NP can be detected using Western blot or immunofluorescence analyses.

In experiments to assess the effect of NEAT1 on the control of hantaviruses, In-Cell Western (ICW) analysis was applied to qualify HTNV NP production. Alterations in the relative fluorescence intensity of NP after silencing or overexpressing NEAT1-2 did not seem to be as remarkable as qRT-PCR or Western blot analysis results. The NP spotted and exhibited in the ICW results forms obvious stains that mimic PFU. However, the specific values scanned and analyzed by the ICW assay reflect only the fluorescence intensity of the integral well instead of the number of spots. As a consequence, the intensity represented the quantity of NP production but could not directly indicate the virulence, which was better shown by plaque-forming assays. The RNAi studies *in vivo* are encouraging (Fig. 8), but the NC used by our group was not mutated si-NEAT1-2 (i.e., same sense strand, but with a point mutation in the targeting strand). The results would be more compelling if the control mice had been treated with the mutated si-NEAT1-2.

One major finding of our study is that the lncRNA NEAT1 serves as positive feedback for RIG-I signaling. After observing that NEAT1 can regulate IFN expression by HTNV infection, we were interested in the function of NEAT1. We noticed that silencing NEAT1-2 or ectopically expressing NEAT1-2 could not inhibit or enhance IFN expression without HTNV infection (Fig. 3F), which indicated that NEAT1-2 could not directly affect IFN-β expression. This finding excludes the possibility that NEAT1-2 directly promoted IFN-β and that IFN-β promoted the expression of PRRs such as RIG-I. Thereafter, NEAT1 was found to modulate HTNV-induced RIG-I and DDX60 expression. Recent findings have shown that RIG-I signaling is essential for an efficient polyfunctional T cell response during IAV infection (42). Indeed, we found that the function of T cells was suppressed after NEAT1-2 depletion in our animal experiments (Fig. 8G), which might be due to the disrupted RIG-I signaling in NEAT1-2 silenced T cells.

**FIG 9:**
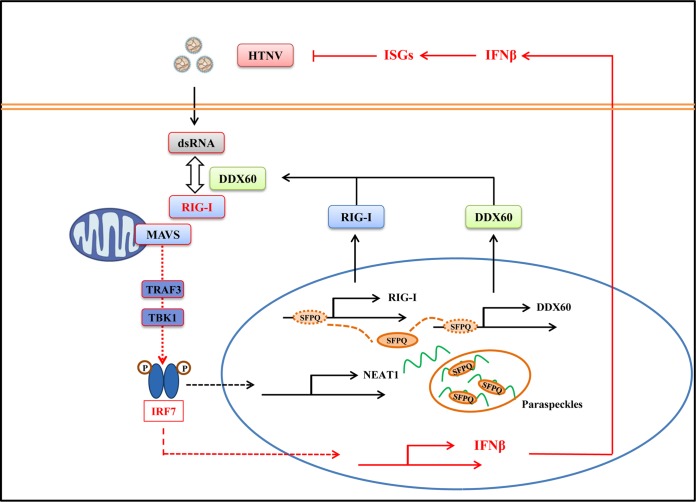
NEAT1 exerts antihantaviral effects by acting as positive feedback for RIG-I signaling. Innate immunity is the first line of defense against microbe invasion. Host PRRs such as RIG-I can recognize the PAMPs of viruses and induce robust innate immune responses against viral infection. Known signaling is shown in boxes outlined in red. Our research indicated that RIG-I and DDX60 might interact with each other to recognize HTNV, which, on the one hand, promotes IFN and cytokine production, and, on the other hand, induces NEAT1 transcription through the RIG-I–IRF7 pathway. As positive feedback, NEAT1 promoted RIG-I and DDX60 expression and enhanced HTNV-induced IFN responses. The specific molecular mechanism might be that NEAT1 relocates SFPQ from the potential promoter region to paraspeckles in the nucleus, thereby facilitating the expression of multiple antiviral genes. Taken together, the lncRNA NEAT1 can exert antihantaviral effects by modulating host innate immune responses.

In conclusion, this is the first study to describe the role of NEAT1 in HTNV infection. HTNV infection induced NEAT1 expression through the RIG-I–IRF7 pathway, while NEAT1 displayed positive feedback for RIG-I signaling. NEAT1 relocated SFPQ from the potential promoter region of several antiviral genes to the paraspeckles, removing the transcriptional inhibitory effects of SFPQ. This phenomenon would facilitate the expression of DDX60 and RIG-I, thus promoting IFN responses and suppressing HTNV infection (Fig. 9).

## MATERIALS AND METHODS

### HTNV strain and virus infection.

HTNV strain 76-118 was propagated in Vero E6 cells (a green monkey kidney epithelial cell line; ATCC no. CRL-1586), and the titers were measured by ELISA as previously described (43). For infection, cells with a confluence of 70 to 80% in 6, 24, or 96 wells or 35-mm dishes were rinsed twice with Dulbecco's phosphate-buffered saline (DPBS; HyClone, Logan City, UT), followed by the addition of HTNV that had been diluted to the desired MOI. After incubation for 90 min at 37°C, the supernatant was discarded. The cells were then washed twice with DPBS, and complete medium was added. As a control, cells were incubated with culture supernatant from uninfected Vero E6 cells, which were referred to as mock-infected cells.

### Cell culture.

A549, HEK293, HeLa, Huh7, and Huh7.5 (RIG-I^−^) cells were purchased from the China Center for Type Culture Collection (CCTCC, Wuhan, China). EVC-304 and EVC-304 TLR4^−^ cells were provided by the Center of Infectious Diseases, Tangdu Hospital (Xi'an, China) (15). A549, HEK293, HeLa, Huh7, Huh7.5 (RIG-I^−^), EVC-304 and EVC-304 TLR4^−^ cells were cultured in Dulbecco's modified Eagle's medium (DMEM; HyClone) with 10% fetal calf serum (HyClone) and 0.1% penicillin-streptomycin solution (HyClone) at 37°C with 5% CO_2_. HUVECs were purchased from ScienCell Research Laboratories (Carlsbad, CA, USA) and cultured in an ECM BulletKit (ScienCell Research Laboratories).

### Transfection for RNAi and overexpression.

siRNAs or plasmids were transfected into cells by using Lipofectamine 2000 (Invitrogen, Carlsbad, CA, USA) or the Amaxa HUVEC Nucleofector kit (Lonza Group Ltd., Basel, Switzerland) according to the manufacturers' instructions. RNA inhibition sequences were synthesized in GenePharma (Shanghai, China). The siRNA sequences targeting NEAT1, NEAT1-2, TLR3, TLR4, SFPQ, NONO, and IRF7 were described previously (11, 44). Notably, si-NEAT1 targets both NEAT1-1 and NEAT1-2, while the stealth RNA NEAT1-2 (st-NEAT1-2) is specific for NEAT1-2. The sequences of nontarget control (i.e., negative control [NC]) or targeted RIG-I and DDX60 were designed by GenePharma as follows: NC, 5′-UUCUUCGAACGUGUCACGUTT-3′; si-RIG-I-1, 5′-GCCCAUUUAAACCAAGAAATT-3′, si-RIG-I-2, 5′-GGUGGAGGAUAUUUGAACUTT-3′, and si-RIG-I-3, 5′-CCCAACGAUAUCAUUUCUTT-3′; si-DDX60-1, 5′-GUCCAGGUGUCAGUUUGAUTT-3′, si-DDX60-2, 5′-CCGAAGUGAAGAAGGUAAATT-3′, and si-DDX60-3, 5′-GAUGGAUGCUAGGAAAUAUTT-3′.

The pCMV-NEAT1-1 and pCMV-NEAT1-2 plasmids, which transcribe NEAT1-1 and NEAT1-2, respectively, were provided by Nakagawa Shinichi (10). The Flag-RIG-I and pUNO-DDX60 plasmids were purchased from Invitrogen.

### Reagents.

Mouse monoclonal antibody (MAb) 1A8 for the HTNV nucleocapsid protein (NP) was produced as previously described (43). Abs against RIG-I, IRF3, IRF7, p65, and STAT1, as well as the neutralizing antibodies against IFN-α and IFN-β, were purchased from Abcam (Cambridge, MA, USA). Phorbol myristate acetate (PMA) and Ab against DDX60 were purchased from Sigma-Aldrich, Inc. (St. Louis, MO, USA). Abs against SFPQ, NONO, GAPDH (glyceraldehyde-3-phosphate dehydrogenase), and β-actin were purchased from Protein Tech, Inc. (Wuhan, China). The Abs targeting CD11b, F4/80, CD3, CD8, IFN-γ, inducible nitric oxide synthase (iNOS), and CD206 for flow cytometry were purchased from BD Biosciences (San Jose, CA, USA). IFN-α, -β, and -γ, TNF-α, and IL-1β were from PeperoTech (Rocky Hill, NJ). ELISA kits for IFN-β detection were manufactured by R&D Systems, Inc. (Minneapolis, MN, USA).

### RNA extraction and quantitative real-time PCR (qRT-PCR) analysis.

Total cellular RNAs were extracted with RNAiso (TaKaRa, Dalian, China), the concentration of which was measured using a NanoDrop 1000 spectrophotometer. Reverse transcription (RT) was then performed with PrimeScript RT master mix (TaKaRa) according to the instructions provided by the manufacturer. Each cDNA was denatured at 95°C for 5 min and amplified for 40 cycles of 15 s at 98°C, 30 s at 58°C, and 30 s at 72°C using a LightCycler 96 (Roche, Basel, Switzerland). The mRNA expression level of each target gene was normalized to the respective β-actin and analyzed. The qRT-PCR primer sequences for NEAT1, NEAT1-2, IFN-β, HTNV S segment, RIG-I, DDX60, β-actin, and GAPDH were obtained from previous reports (24, 45). The methods used to quantify HTNV RNA load have been described by our group previously (46).

### DGE analysis and lncRNA sequencing.

HUVECs with a confluence of 80% in 6 wells were mock infected or infected with live or ^60^Co-inactivated HTNV at an MOI of 1. RNAs were extracted as previously described at 24 hpi, and the quality was analyzed using FastQC software by the Beijing Genomics Institute (BGI, Shenzhen, China). Digital gene expression (DGE) tags were annotated to the human transcriptome (Ensembl version 58) by mapping the reads to the sequence flanking NlaIII restriction sites on both coding and noncoding strands. Tags matching more than one gene region were discarded. Tag counts were normalized to TPM (transcripts per million) by dividing the raw tag count by the total number of tags from each library and multiplying by 1 million. To avoid the possible noise signal from high-throughput sequencing, the genes with average TPM of less than 1 in these three states were excluded. In this study, an absolute fold change of no less than 1.5 and a false discovery rate (FDR) of less than 0.001 were used to define the differentially expressed genes. Genes were selected as differentially expressed using a *P* value threshold of 0.01. Genes were selected as differentially expressed using a *P* value threshold of 0.01.

### FISH and immunofluorescence assays (IFA).

Fluorescence *in situ* hybridization (FISH) was performed with a FISH kit (Ribobio Co.) according to the manufacturer's instructions. In brief, cells were fixed with 4% paraformaldehyde (PFA) for 10 min at room temperature and permeabilized with 0.5% Triton X-100 for 15 min at room temperature. Prehybridization was performed with lncRNA FISH probe mix at 37°C for 30 min, and then hybridization was performed by adding NEAT1-2 FISH probe mix and incubating the mixture at 37°C overnight. After washing with 4×, 2×, and 1× SSC, the cell nuclei were stained with DAPI (4′,6-diamidino-2-phenylindole). Finally, the samples were observed using a BX60 fluorescence microscope (Olympus, Tokyo, Japan).

IFA was performed after FISH or independently. The cells were fixed with 4% PFA for 10 min and permeabilized with 0.1% Triton X-100 for 15 min. Primary Abs were added and incubated at 37°C for 2 h. After five washes with DPBS, secondary Cy3- or fluorescein isothiocyanate (FITC)-conjugated goat anti-rabbit or goat anti-mouse IgG (Sangon, Shanghai, China) was added and incubated at 37°C for 2 h. Cell nuclei were stained with DAPI. Finally, the samples were observed using a BX60 fluorescence microscope (Olympus).

### SDS-PAGE and Western blot analysis.

Cells were washed twice with ice-cold DPBS and lysed with 1× SDS protein loading buffer (50 mM Tris, 2% SDS, 10% glycerol, 2% 2-mercaptoethanol, and 0.1% bromophenol blue). The samples were then boiled at 95°C for 10 min. The lysates were resolved by 10%, 12%, or 15% SDS-PAGE and transferred to polyvinylidene fluoride (PVDF) membranes (Millipore). The membranes were incubated with the primary antibodies, followed by secondary antibodies labeled with infrared dyes (Li-Cor Biosciences, Lincoln, NE, USA). The signals on the PVDF membrane were visualized using an Odyssey infrared imaging system (Li-Cor Biosciences, Lincoln, NE, USA).

### ICW assay.

The In-Cell Western (ICW) assay was performed using an Odyssey imaging system (Li-Cor) according to the manufacturer's instructions. HUVECs were either mock transfected or transfected with NC sequences, si-NEAT1, st-NEAT1-2, vector plasmids, pCMV-NEAT1-1, or pCMV-NEAT1-2 and grown in 96-well plates (2 × 10^4^ cells/well). Twenty-four hours posttransfection, the cells were either infected or mock infected with HTNV at an MOI of 1. At 48 hpi, HUVECs were washed twice with ice-cold DPBS, fixed with 4% PFA for 10 min, and permeabilized with 1.0% Triton X-100 for 15 min. Cells were added with Li-Cor Odyssey blocking solution at room temperature for 30 min and incubated at 4°C overnight with mouse IgG MAb 1A8 against HTNV NP together with rabbit IgG antibody against β-actin, both of which were diluted in PBS containing 3% bovine serum albumin (BSA; HyClone). Subsequently, the cells were washed and stained with goat anti-mouse IgG IRDye 800 antibody (1:5,000; Li-Cor) and goat anti-rabbit IgG IRDye 680 antibody (1:5,000; Li-Cor) at room temperature for 2 h. The plates were scanned with the Odyssey imaging system, and the integrated intensities of fluorescence indicating the protein expression levels were acquired using the software provided with the imager station (Li-Cor). The relative amount of NP protein was obtained by normalization to the endogenous β-actin in all experiments.

### Co-IP.

Coimmunoprecipitation (Co-IP) RIP kits were purchased from Protein Tech, Inc. HEK293 cells were mock infected or infected with HTNV at an MOI of 1 and lysed with IP lysis buffer at 48 hpi. The lysates were incubated with rabbit anti-SFPQ polyclonal antibody and incubation buffer at 4°C for 4 h in spin columns. Meanwhile, control IgG was added to the lysate as a negative control in the mock and HTNV infection group. Protein A Sepharose beads were then added, incubated at 4°C overnight, and then eluted with elution buffer. After centrifugation at 1,000 rpm at 4°C for 10 min, alkali neutralization buffer and 1× sample buffer were added to the elution product. The IP samples were stored at −80°C for Western blot analysis.

### RIP.

RNA immunoprecipitation (RIP) kits were purchased from Millipore (Darmstadt, Germany). HUVECs were infected with HTNV at an MOI of 1 and collected for RIP at 0, 1, 2, and 3 dpi. RIP was performed according to the manufacturer's protocol. In brief, cells were lysed in polysome lysis buffer and immunoprecipitated with Abs against SFPQ with protein A/G magnetic beads. Magnetic bead-bound complexes were immobilized with a magnet, and unbound material was removed by washing. Finally, RNAs were extracted and stored at −80°C for further qRT-PCR analysis.

### Animal grouping and handling.

Seven-week-old male C57BL/6 mice (18 to 22 g) were provided by the Experimental Animal Center of the Fourth Military Medical University (FMMU). Experiments with mice were conducted in compliance with a protocol approved by the Institutional Animal Care and Use Committee of FMMU based on the Ethical Principles in Animal Experimentation. Eighty C57BL/6 mice were randomly assigned to PBS, PBS+HTNV, NC+HTNV, and si-NEAT1-2+HTNV groups (*n* = 20 in each group). si-NEAT1-2 (10 μg) designed and synthesized by GenePharma (5′-CAGCUGUACUGUGAGAUCAUGUUCU-3′) was mixed with 10 μl Lipofectamine and injected intravenously in the si-NEAT1-2+HTNV group every 3 days; the other animals were injected with either PBS or negative-control sequence (NC; 5′-UUCUUCGAACGUGUCACGUTT-3′). The mice were administered a median lethal dose (LD_50_) of HTNV 2 days after pretreatment. Mouse body weight was measured from 0 to 10 dpi. At 2 dpi, 6 mice were sacrificed, and livers, spleens, and kidneys were collected to measure alterations in NEAT1 by qRT-PCR. At 3 dpi, 8 mice were sacrificed, and livers, spleens, and kidneys were collected to detect HTNV NP by ELISA or HTNV S segment levels by qRT-PCR. In addition, organs soaked in RPMI 1640 were subjected to three freeze-thaw cycles to evaluate the HTNV titers. At 7 dpi, 4 mice in each group were anesthetized for blood collection from the heart, sera from which were prepared to detect the titers of neutralizing antibody against HTNV by ELISA (47).

### Statistical analysis.

All data were expressed as the mean ± standard error of the mean (SEM). Statistical analyses were performed using GraphPad Prism 5 (GraphPad Software, La Jolla, CA, USA). Independent sample *t* tests were used for normally distributed data. Differences among groups were determined by one-way analysis of variance (ANOVA) with repeated measures, followed by Bonferroni's *post hoc* test. A *P* of <0.05 was considered statistically significant.

### Accession number(s).

DGE data are available online at the Gene Expression Omnibus (GEO) under accession number GSE89950.
